# Expression Profiling of Preadipocyte MicroRNAs by Deep Sequencing on Chicken Lines Divergently Selected for Abdominal Fatness

**DOI:** 10.1371/journal.pone.0117843

**Published:** 2015-02-12

**Authors:** Weishi Wang, Zhi-Qiang Du, Bohan Cheng, Yuxiang Wang, Jing Yao, Yumao Li, Zhiping Cao, Peng Luan, Ning Wang, Hui Li

**Affiliations:** Key Laboratory of Chicken Genetics and Breeding, Ministry of Agriculture, Key Laboratory of Animal Genetics, Breeding and Reproduction, Education Department of Heilongjiang Province, College of Animal Science and Technology, Northeast Agricultural University, Harbin, P.R. China; South China Agricultural University, CHINA

## Abstract

Through posttranscriptional gene regulation, microRNA (miRNA) is linked to a wide variety of biological processes, including adipogenesis and lipid metabolism. Although miRNAs in mammalian adipogenesis have been worked on extensively, their study in chicken adipogenesis is still very limited. To find miRNAs potentially important for chicken preadipocyte development, we compared the preadipocyte miRNA expression profiles in two broiler lines divergently selected for abdominal fat content, by sequencing two small RNA libraries constructed for primary preadipocytes isolated from abdominal adipose tissues. After bioinformatics analyses, from chicken miRNAs deposited in miRBase 20.0, we identified 225 miRNAs to be expressed in preadipocytes, 185 in the lean line and 200 in the fat line (derived from 208 and 203 miRNA precursors, respectively), which corresponds to 114 miRNA families. The let-7 family miRNAs were the most abundant. Furthermore, we validated the sequencing results of 15 known miRNAs by qRT-PCR, and confirmed that the expression levels of most miRNAs correlated well with those of Solexa sequencing. A total of 33 miRNAs was significantly differentially expressed between the two chicken lines (P<0.05). Gene ontology analysis revealed that they could target genes enriched in the regulation of gene transcription and chromatin function, response to insulin stimulation, and IGF-1 signaling pathways, which could have important roles in preadipocyte development. Therefore, a valuable information and resource of miRNAs on chicken adipogenesis were provided in this study. Future functional investigations on these miRNAs could help explore related genes and molecular networks fundamental to preadipocyte development.

## Introduction

As small non-coding RNA molecules, ~22 nucleotides in length, microRNAs (miRNAs) can regulate their RNA targets by either direct degradation or translational inhibition through partial complementary sequence recognition and binding. Detailed studies on miRNA genomics, evolution and function revealed that they are involved in a wide variety of biological processes, such as cell differentiation, proliferation, disease development [1,2], as well as adipogenesis and lipid metabolism [3–23].

Adipogenesis is orchestrated by a fine balance of molecular and cellular signals, the disruption of which could change adipocyte size or number, and the ensuing expansion or contraction of white adipose tissue will happen [3]. In mammals, a number of miRNAs have been demonstrated to target genes involved in adipogenesis and lipid metabolism, such as the regulation on the proliferation of adipose tissue-derived mesenchymal stem cells by miR-21 and miR-196a [4–6]; the enhancement of adipogenesis by miR-103, miR-224 and the miR-17–92 cluster [7–9]; the impairment of adipogenesis by the let-7 family, miR-448, miR-15a and miR-27 [10–13]; the regulation of adipocyte lipid metabolism by miR-27a and miR-143 [13–15]; and the important role of miR-33 on the repression of sterol transporters reported in numerous studies [16–24].

In chickens, a majority of the miRNA studies have been performed to examine their roles in growth performance, reproduction and disease resistance [25–31]. However, very few studies for chicken adipogenesis were conducted. One study examined the role of miR-33 on the regulation of FTO gene, which is important in adipose tissue development [24]. Two other studies were limited in the identification of miRNAs, one in muscle and adipose tissues [32], and the other in preadipocytes obtained from Arbor Acres (AA) broilers reported previously by our group [33].

In order to better understand miRNAs involved in chicken adipogenesis, we used the Northeast Agricultural University broiler lines divergently selected for abdominal fat content (NEAUHL), which showed marked difference in abdominal fat content between the two lines, and have been studied extensively in searching for genetic factors underlying the development of adipose tissue [34–36]. Small RNA libraries were constructed and sequenced, using primary preadipocytes collected from abdominal fat tissues. After comparison between the fat and lean broiler lines, a total of 33 miRNAs were found to be significantly differentially expressed. Furthermore, gene ontology analyses showed that the target genes of these differentially expressed miRNAs were enriched in pathways potentially related to adipocyte development and lipid metabolism, such as transcription regulation, chromatin regulator, response to insulin stimulation, and more interestingly, IGF-1 signalling pathway and epigenetic regulation of gene expression. We found that most of these miRNAs could be important to adipogenesis, by extensive literature mining and a combined analysis of gene expression profiling on chicken preadipocytes. Future investigation on the relationship between the function of these miRNAs and preadiopocyte development is still warranted, which could help explore related genes and molecular networks fundamental to preadipocyte development.

## Results

We sequenced two small RNA libraries built from lean and fat broiler preadipocytes, which contained 14,146,164 and 15,723,681 raw reads, respectively. In the lean chicken line, 80.60% of total reads and 53.65% of unique reads could be mapped to the chicken reference genome, and for the fat chicken line, the proportions were 82.96% and 46.68%, respectively.

**Fig 1:**
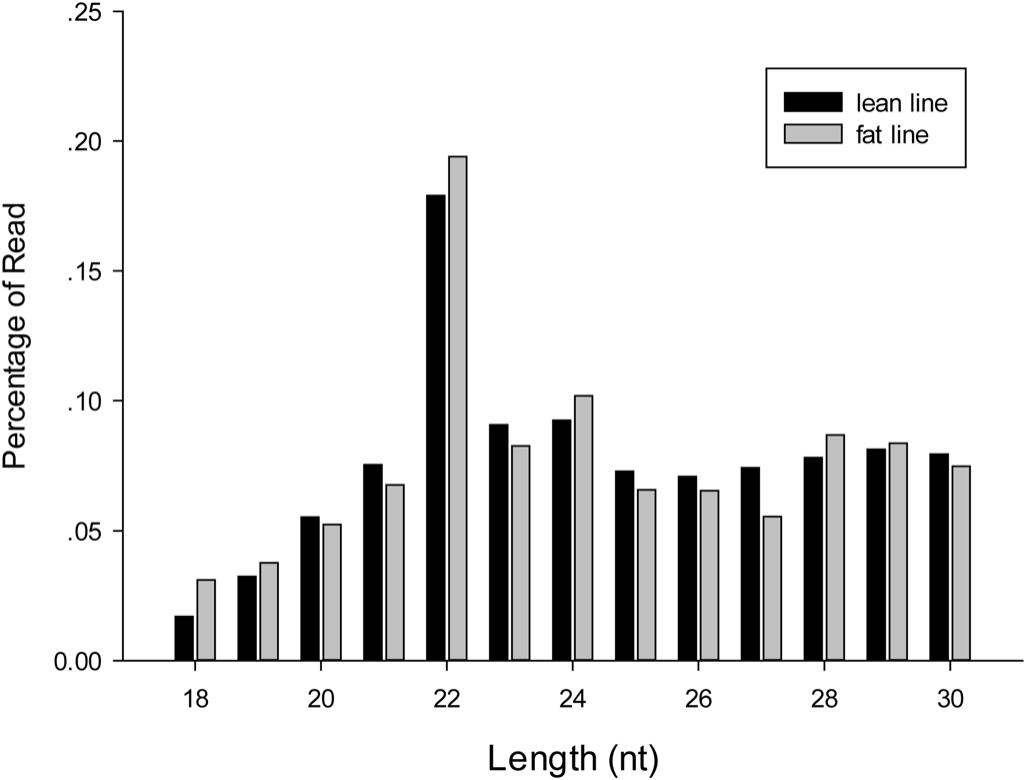
Length distribution of small RNA read sequences in fat and lean chicken lines.

After quality control procedures (see Methods), 13,463,693 and 12,490,340 short reads were kept for further analyses for the lean and fat chicken lines, respectively (Table 1). We found that 8.58% and 8.45% of the clean reads correspond to miRNAs for the lean and fat chicken lines, respectively. The remaining clean reads could be mapped to other genomic locations, corresponding to other different kinds of small RNAs, including repeats, snRNA, snoRNA, rRNA and tRNA (S1 Table). In agreement with the definition of miRNA, it is clear that in the length range of 18 to 30 nucleotides (nt), reads of 22 nt are the most abundant, distributed with a percentage of 16% and 18% in the lean and fat chicken lines, respectively (Fig. 1).

**Table 1:** Summary of short reads produced by small RNA sequencing after data cleaning procedure in each sample.

Type	Lean line		Fat line	
	Count	Percentage	Count	Percentage
**3′-adapter**	127,283	0.92	418,321	2.70
**Insert**	86,270	0.62	1,719,912	11.12
**5′-adapter**	104,238	0.75	140,980	0.91
**<18 nt**	73,063	0.53	698,054	4.51
**polyA**	698	0.01	899	0.01
**Clean reads**	13,463,693	97.17	12,490,340	80.75
**High-quality reads**	13,855,245	100	15,468,506	100
**Total reads**	14,146,164		15,723,681	

### Identification of miRNAs in the two chicken lines

**Fig 2:**
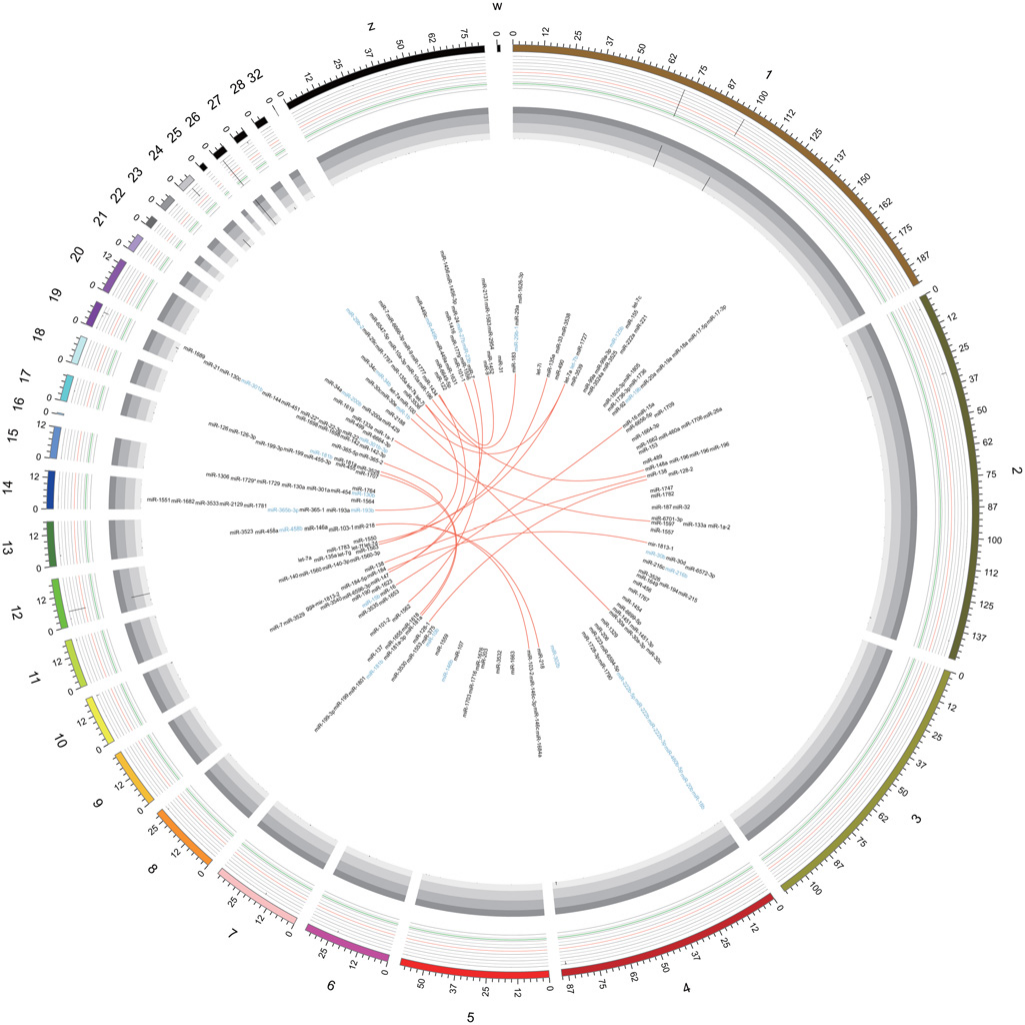
Circular view of miRNAs identified in the chicken genome. Data tracks viewed from outside inwards: 1) chicken chromosomes; 2) and 3) miRNAs abundantly expressed in the lean and fat chicken lines, respectively; 4) miRNA labels; 5) link lines for miRNA paralogs found to be expressed in chicken preadipocytes. Details on the sequence alignment between miRNA precursors for these miRNA paralogs (chromosome coordinates and sequence identity) can be found in S2 Table.

**Fig 3:**
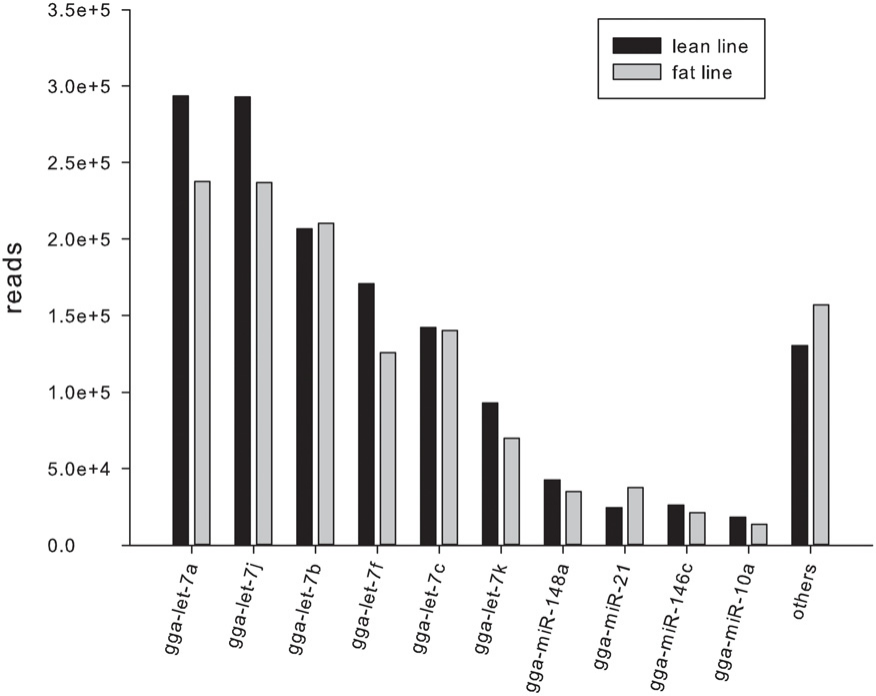
The top 10 most abundantly expressed miRNAs.

Of the 996 *Gallus gallus* mature miRNAs deposited in miRBase 20.0, 225 were identified in our two libraries (185 in the lean line and 200 in the fat line) (Fig. 2), which belonged to a total of 114 miRNA families, and were derived from 208 and 203 miRNA precursors (pre-miRNAs) in the lean and fat lines, respectively. The top 10 abundant miRNAs included the let-7 miRNA family (let-7a, j, b, f, c, and k), gga-miR-148a, gga-miR-146c, gga-miR-10a, and gga-miR-21. The let-7 miRNA family was the most abundant, representing 83.3% and 79.46% of the total reads in lean and fat broilers, respectively. Moreover, gga-let-7a and gga-let-7j were the most frequently sequenced miRNA in both lines (>18%) (Figs. 2 and 3). Three other miRNAs, gga-miR-148a, gga-miR-146c and gga-miR-10a were more abundant in the lean line than in the fat line. In contrast, gga-miR-21 was more abundant in the fat line.

**Fig 4:**
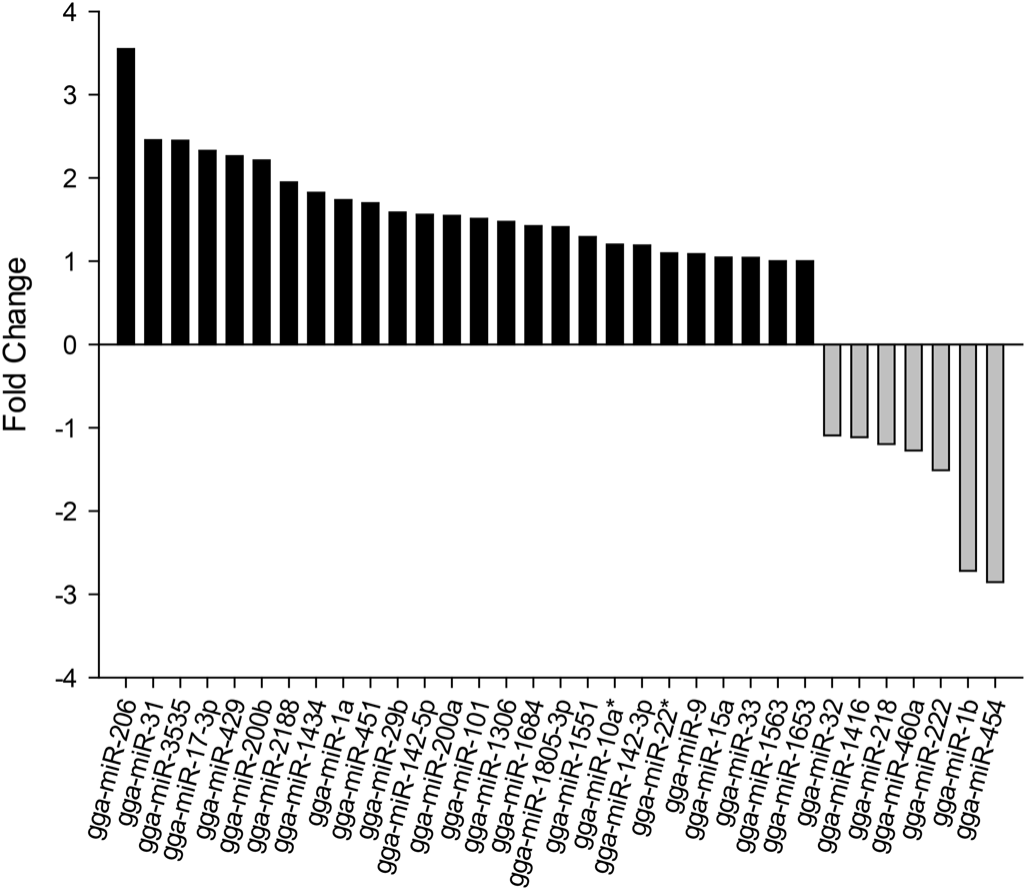
Differentially expressed miRNAs. 26 up-regulated miRNAs, and 7 down-regulated miRNAs in the fat chicken line. Fold-change (Y-axis) indicates in log2-scale the fold-changes between the number of reads of miRNAs in the fat chicken lines and the lean chicken line.

Among the 225 miRNAs identified, 33 were significantly differentially expressed between the two chicken lines (P<0.05), in which 26 miRNAs were highly expressed (up-regulated), and 7 miRNAs were lowly expressed (down-regulated) in the fat chicken line. The most significantly differentially expressed were gga-miR-206 (3.5-fold), gga-miR-31 (2.5-fold), gga-miR-3535 (2.5-fold), gga-miR-17–3p (2.3-fold), gga-miR-429 (2.3-fold) and gga-miR-200b (2.2-fold), and in comparison, gga-miR-454 (-2.9-fold) and gga-miR-1b (-2.7-fold) were those mostly down-regulated in the fat line (Fig. 4). Among the 33 significantly differentially expressed miRNAs, gga-miR-101 had the largest number of reads in both fat and lean lines.

In addition, in searching for novel miRNAs, we adopted the three-step approach, including the control on number of short reads, prediction of hairpin structure, and constraints on both number of short reads and locations corresponding to novel miRNAs (details as in Methods). A total of 107 and 571 novel miRNAs were found in the lean and fat chicken lines, respectively (S3 Table and S4 Table). Only were three novel miRNAs found to be in common in both lines (gga01:118610977–118611076, gga11:552873–552951, and gga26:640852–640926).

### Verification of sequencing results

**Fig 5:**
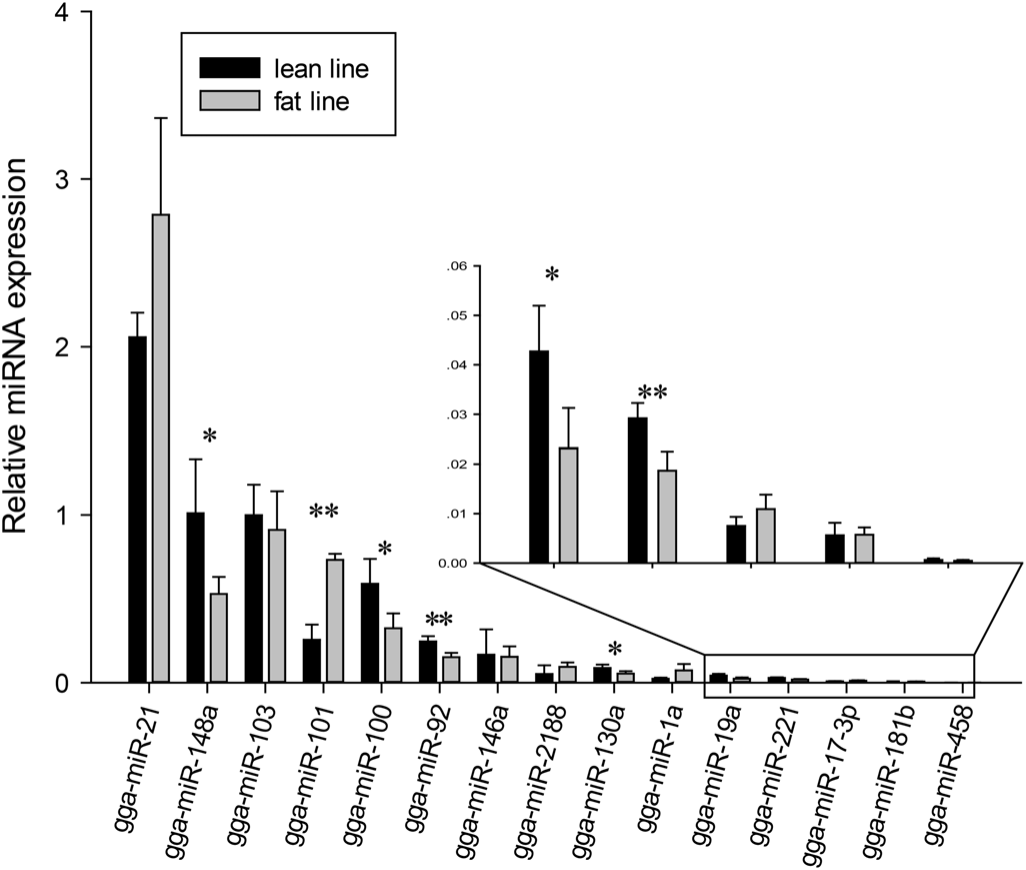
qRT-PCR validation of miRNAs in the preadipocytes of the lean and fat chicken lines. miRNA expression levels were normalized, and were selected at high, intermediate and low levels according to their number of reads by Solexa sequencing. Seven miRNAs (gga-miR-148a, gga-miR-101, gga-miR-100, gga-miR-92, gga-miR-130a, gga-miR-19a and gga-miR-221) with significantly differential expression levels were found (* P<0.05; ** P<0.01). Inset shows the enlarged view of the five lowly expressed miRNAs.

To validate the results obtained from high-throughput sequencing, we selected 15 miRNAs already known in miRBase and 22 novel miRNAs detected in this study for validation by qRT-PCR, respectively. Firstly, for the 15 known miRNAs, we extracted RNA from primary preadipocytes isolated from abdominal tissues for a total of 10 birds (5 from each of the two NEAUHL chicken lines at 16^th^ generation), and each bird was assayed individually and in triplicate. These miRNAs were divided into three groups according to their expression levels in the fat broiler line, 4 highly expressed (gga-miR-21, gga-miR-148a, gga-miR-103, gga-miR-101) (2^-ΔCt^ >0.7), 4 moderately expressed (gga-miR-100, gga-miR-146a, gga-miR-92, gga-miR-2188) (0.7>2^-ΔCt^>0.08), and 7 lowly expressed (gga-miR-1a, gga-miR-130a, gga-miR-221, gga-miR-19a, gga-miR-181b, gga-miR-458, gga-miR-17–3p) (2^-ΔCt^<0.08) (Table 2). Four miRNAs significantly differentially expressed between the fat and lean chicken lines detected by deep sequencing were included in the list, i.e. gga-miR-101, gga-miR-2188, gga-miR-1a and gga-miR-17–3p. After qRT-PCR analyses, gga-miR-101 was also found to be significantly differentially expressed, and gga-miR-1a and gga-miR-17–3p were suggestively significant. Consequently, our qRT-PCR analyses confirmed that the expression level of most of the 15 miRNAs correlated well with those of Solexa sequencing (S1 Fig.). Furthermore, we found that two miRNAs, gga-miR-92 and gga-miR-221, were significantly differentially expressed between the two chicken lines by qRT-PCR analyses. The discrepancy of the significance of miRNA expression between the two lines could be possibly due to that animals from different generations were assayed, and individually (see Methods) (Fig. 5).

**Table 2:** Number of reads and expression levels of the 15 miRNAs chosen for qRT-PCR validation.

	Number of reads in the lean line	2^-ΔCt^ in the lean line (Mean±SD)	Number of reads in the fat line	2^-ΔCt^ in the fat line (Mean±SD)	p-value
**gga-miR-21**	2115	2.06±0.15	3391	2.79±0.58	0.06
**gga-miR-148a**	3326	1.01±0.32	3000	0.53±0.10	0.04
**gga-miR-103**	875	0.99±0.18	1289	0.91±0.23	0.56
**gga-miR-101**	446	0.26±0.09	1272	0.73±0.04	0.0001
**gga-miR-100**	373	0.59±0.15	215	0.32±0.09	0.02
**gga-miR-92**	78	0.25±0.03	99	0.15±0.03	0.002
**gga-miR-146a**	105	0.17±0.15	158	0.15±0.06	0.89
**gga-miR-2188**	27	0.05±0.05	104	0.09±0.03	0.20
**gga-miR-130a**	55	0.09±0.02	71	0.05±0.01	0.03
**gga-miR-1a**	36	0.02±0.01	122	0.07±0.04	0.06
**gga-miR-19a**	15	0.04±0.01	18	0.02±0.01	0.01
**gga-miR-221**	25	0.03±0.003	37	0.02±0.004	0.003
**gga-miR-17–3p**	4	0.008±0.002	22	0.01±0.003	0.09
**gga-miR-181b**	6	0.006±0.003	6	0.006±0.001	0.93
**gga-miR-458**	6	0.001±0.0003	2	0.0004±0.0002	0.39

**Fig 6:**
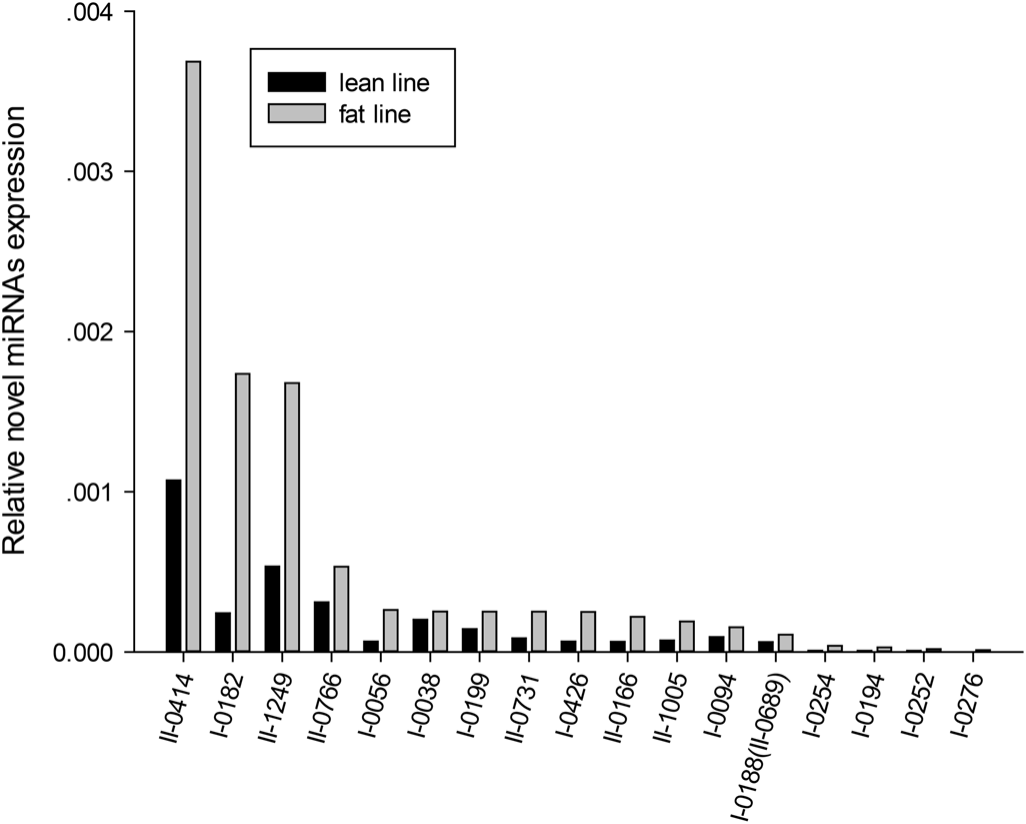
Expression validation of 17 novel miRNAs. Novel miRNAs have relative low expression levels, consistent with their low read numbers by Illumina sequencing.

Secondly, for the 22 novel miRNAs selected for validation, we found that 17 of them (77.27%) did exist. These 17 miRNAs expressed with relatively low abundance, which was in accordance with the low number of short reads in the miRNA sequencing data (Fig. 6, Table 3).

**Table 3:** Expression level of 17 novel miRNAs validated.

	Number of reads in the lean line	2^-ΔCt^ in the lean line	Number of reads in the fat line	2^-ΔCt^ in the fat line	Fold-change*
II-0414	0	1.07E-03	86	3.68E-03	3.44
I-0182	47	2.42E-04	0	1.74E-03	7.19
II-1249	0	5.33E-04	35	1.68E-03	3.15
II-0766	0	3.10E-04	30	5.32E-04	1.72
I-0056	52	6.51E-05	0	2.63E-04	4.04
I-0038	10	2.02E-04	0	2.53E-04	1.25
I-0199	155	1.43E-04	0	2.51E-04	1.76
II-0731	0	8.57E-05	10	2.51E-04	2.93
I-0426	37	6.54E-05	0	2.50E-04	3.82
II-0166	0	6.35E-05	15	2.20E-04	3.46
II-1005	0	7.17E-05	68	1.91E-04	2.66
I-0094	23	9.30E-05	0	1.54E-04	1.66
I-0188(II-0689）	57	6.15E-05	12	1.08E-04	1.76
I-0254	20	8.53E-06	0	3.89E-05	4.56
I-0194	68	7.90E-06	0	2.92E-05	3.70
I-0252	220	7.73E-06	0	1.70E-05	2.20
I-0276	66	3.82E-06	0	1.23E-05	3.22

Note: * indicates the fold changes of 2^-ΔCt^ values in the fat line against the lean line.

### Gene ontology analyses

We searched the target genes of the 33 significantly differentially expressed miRNAs. In total, 2,097 and 1,212 genes were found to be targeted by the 26 up-regulated miRNAs and the other 7 down-regulated miRNAs in the fat broiler line, respectively. Within these two sets of genes, 853 were common.

**Fig 7:**
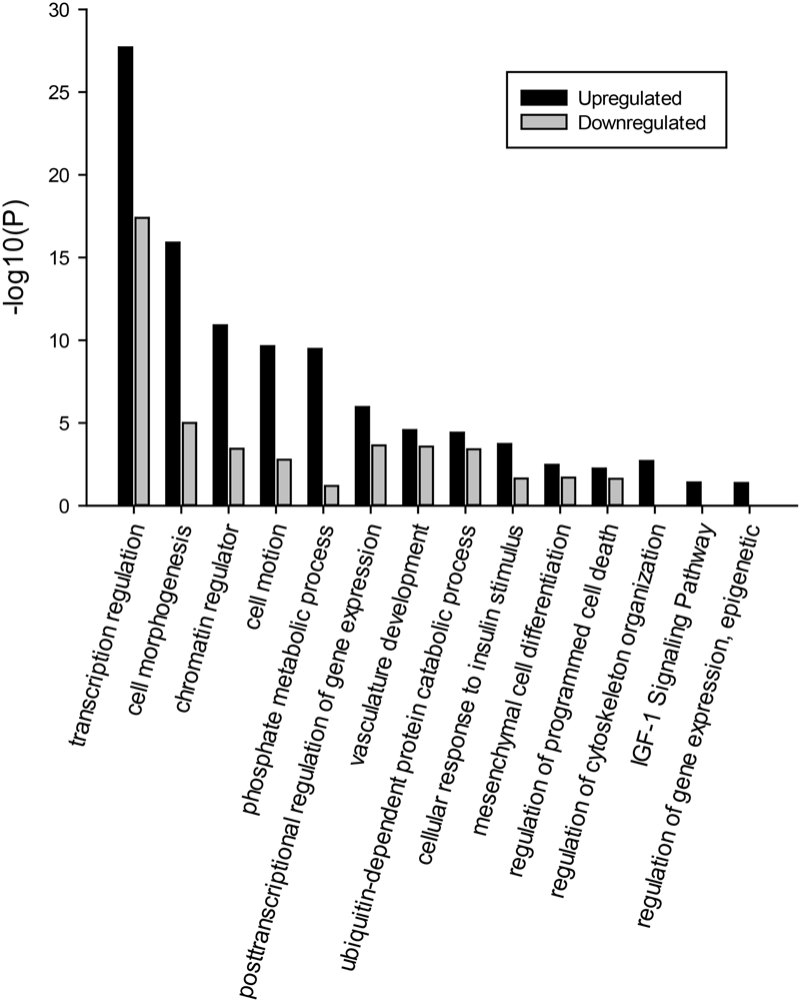
Enriched biological pathways of genes targeted by 33 up-regulated and down-regulated miRNAs with significantly differential expression level. Y-axis shows the significance level of enrichment after Bonferroni adjustment.

Gene ontology analyses were performed by the DAVID approach on all the 2,456 genes targeted by the 33 significantly differentially expressed miRNAs. Genes predicted to be targeted by those 33 differentially expressed miRNAs were significantly enriched in several biological processes, including transcription regulation, chromatin regulator, cell morphogenesis, cellular response to insulin stimulus, mesenchymal cell differentiation, and the regulation of programmed cell death (Fig. 7). Furthermore, the separate gene ontology analyses identified three interesting biological pathways, i.e. cytoskeleton regulation, IGF-1 signaling pathway and epigenetic regulation of gene expression, which appeared to be targeted specifically by miRNAs up-regulated in the fat broilers. These biological processes could potentially contribute to explaining the different levels of fatness in the two divergently selected chicken lines.

## Discussion

In the current study, we compared miRNA expression profiles for preadipocytes collected from fat and lean broilers divergently selected for abdominal fat content, and identified 225 known miRNAs. We further validated the sequencing results of 15 known miRNAs by qRT-PCR, and confirmed the expression level of most miRNAs correlated well with those of Solexa sequencing. Gene ontology analysis revealed that 33 significantly differentially expressed miRNAs between the two chicken lines could participate in a variety of biological processes potentially important to preadipocyte development.

Adipocyte differentiation is a complex process requiring fine-tuning of a series of cellular and molecular events orchestrated by transcription factors and other regulatory elements, which can be further complicated by an extra layer of regulation posed upon by epigenetic regulators and endocrine signals [37–39]. Interestingly, our gene ontology analyses on genes targeted by the 33 significantly differentially expressed miRNAs identified several biological processes, including cellular response to insulin stimulus, mesenchymal cell differentiation, transcription regulation and chromatin regulator, cell morphogenesis and the regulation of programmed cell death. It is well recognized that insulin is directly related to the regulation of glucose level, and insulin resistance brings detrimental effects to cellular metabolism, leading to metabolic diseases, e.g. obesity [40–42]. Adipocytes can be differentiated from mesenchymal stem cells, and the regulation on this process and the ensuing cell morphogenesis and cell death pathway will definitely affect the development of adipose tissue [43–45]. Chromatin modification and transcription regulation, such as gene methylation and histone modifications, were also found to be important to adipogenesis [46–48]. Recently, miR-148a was found to be able to target DNMT1, affecting the methylation status of adipocytes [49].

Three biological processes targeted specifically by up-regulated miRNAs in the fat chicken line, cytoskeleton regulation, IGF-1 signalling pathway and epigenetic regulation of gene expression, are *bona fide* molecular pathways demonstrated to be fundamental to adipocyte development. Cytoskeleton remodeling through acetyltransferase MEC-17, BSCL2/seipin and oxygen levels were recently found to be related to adipogenesis [50–52]. IGF-1 has long been regarded as one of the adipocyte differentiation stimulators, and extensive studies have been conducted to study its exact roles on growth, obesity and disease development [53–56]. In chickens, it was shown to be related to growth and abdominal fat content [57,58], and in our divergently selected chicken lines, the genomic region containing IGF-1 on chicken chromosome 1 was also shown to be under selection by selective sweep analysis [35]. The epigenetic effects on gene transcription, and its potential relationship to adipogenesis were also reported previously [46–49].

In addition, we searched the genes targeted by down-regulated miRNAs in the fat broiler lines for enriched biological processes. However, we did not find any interesting significant processes, possibly due to the few number of genes (a total of 105).

After exhaustive literature mining, among the 33 significantly differentially expressed miRNAs, a total of 21 miRNAs have been demonstrated to be involved in (pre-)adipocyte development and metabolism (details in S5 Table). The most interesting miRNA could be miR-33, reported in a number of studies [16–24], which is highly important on cholesterol homeostasis by repressing sterol transporters. For miR-222, a known regulator of kit ligand signaling during the recruitment and maintenance of precursor hematopoietic cells, it has been described to be related to adipose tissue development in mice treated with conjugated linoleic acid [59]. With the highest number of reads in both chicken lines among the significantly differentially expressed miRNAs, miR-101 was found previously to be able to induce the differentiation of 3T3-L1 [60]. As a member of the miR-17–92 cluster, miR-17–3p can target RB and fatty acyl desaturase genes [61,62], and enhance 3T3-L1 differentiation [9]. Through the regulation of DLK1 level, miR-15a could disrupt adipogenic differentiation [12]. miR-15a can also target Foxo1, and participate in the disruption of adipogenic differentiation and the regulation of insulin synthesis [63–65]. miR-22 can regulate the PTEN/AKT pathway and target HDAC6 [66–68]; miR-206 and miR-1a can suppress hepatic lipogenesis [69]; miR-29b and miR-9 are involved in insulin sensitivity and diabetes [70,71]; miR-31 and miR-32 participate in differentiation of stem cells into adipocyte and lipid metabolism in oligodendrocytes [72,73]. Last but not least, expression levels of 10 miRNAs can be perturbed in animals when fed with high-fat diet (miR-142–5p and miR-101) [74–78], or with obesity or obesity-related diseases (miR-10a, miR-218, miR-429, miR-200a, miR-200b, miR-451, miR-142–3p, and miR-454) [77,79–85], which indicates that they could be potentially related to adipogenesis.

However, among the remaining 12 miRNAs, we found that miR-2188 affects embryonic development in fishes [86,87]; miR-1306 is related to Alzheimer’s disease by targeting ADAM10 [88]; miR-1684 was differentially expressed in chicken lines selected for necrotic enteritis [89]; miR-1b could be potentially related to immunity genes in insects [90]; no literature was found for the other 8 miRNAs (miR-3535, miR-1434, miR-1805–3p, miR-1551, miR-1563, miR-1653, miR-1416, miR-460a). Though no direct evidence on their roles in adipogenesis or lipid metabolism was found, we still could not exclude the possibility of their involvement.

We further analyzed gene expression data for birds from a time-course transcriptome profiling project performed previously in our lab (GEO accession number: GSE51330), in which a similar procedure for collecting chicken preadipocytes was used, and both broiler lines were analyzed. As a result, among all genes targeted by the 33 differentially expressed miRNAs, 262 genes significantly differentially expressed had the same trend of expression levels, i.e. genes targeted by up-regulated miRNAs having lower expression levels, and *vice versa* (S2 Fig., S6 Table). After gene ontology analysis, these 262 genes were also found to be significantly enriched in cytoskeleton regulation and chromatin regulator pathways.

In addition, 107 and 571 novel miRNAs were found in the lean and fat chicken lines, respectively. We found 17 of 22 novle miRNAs selected for validation (77.3%) expressed in the same RNA sample used for miRNA sequencing, but all with a relatively low level of expression. In another study, 50 out of 53 novel miRNAs discovered in lancelet were verified by expression analysis, after using the same software MIREAP in predicting novel miRNAs [91]. Furthermore, 18 of 25 (72.0%) novel miRNAs were confirmed in mice with ectopic expression analysis [92]; 23 of 30 (76.6%) were verified in a pig study with direct PCR amplification [93]; 12 of 17 (70.6%) were detected by northern blot analysis in a human study [94]. All these studies have a comparable success rate of novel miRNA validation with ours. We failed to validate 5 novel miRNAs, possibly due to prediction accuracy, or limitation of experimental techniques to detect these novel miRNAs [93,95]. Another possibility could be that the 5 novel miRNAs were simply not real miRNAs, since 2 of them could not be amplified, and the remaining 3 had an irregular melting curve when performing qRT-PCR.

## Conclusions

Taken together, in this study, the expression profiling of abundantly and differentially expressed miRNAs in preadipocytes derived from two divergently selected chicken lines were performed. Some miRNAs such as gga-miR-101 and gga-miR-1a were significantly differentially expressed between the fat and lean chicken lines. After exhaustive literature mining and gene ontology analyses, the 33 differentially expressed miRNAs were found to potentially play important roles in several biological processes, such as chromatin regulator, cell morphogenesis, cellular response to insulin stimulus, mesenchymal cell differentiation, which could be involved in preadipocyte development. Our results will provide valuable resources and information for further functional investigation on the relationship between miRNA function and chicken adipogenesis.

## Materials and Methods

### Ethics statement

Animal work was conducted following the guidelines for the care and use of experimental animals established by Ministry of Science and Technology of People’s Republic of China (approval number: 2006–398), and also approved by Laboratory Animal Management Committee of Northeast Agricultural University.

### Animals and cell culture

Broilers for miRNA expression profiling were selected from the 15^th^ generation population of Northeast Agricultural University broiler lines divergently selected for abdominal fat content (NEAUHL), which showed 4.58 times difference in abdominal fat percentage between the fat and lean lines. Both chicken lines were kept in the same environmental conditions, and had access to feed and water *ad libitum*. Details on the selection program of the two lines can be found in our previous report [34].

Chicken preadipocytes for miRNA sequencing library construction were collected according to previously described methods with a few modifications [96,97], as well as a recent method published by our group [98]. Briefly, abdominal adipose tissue was taken from 12 fourteen-days-old male broilers (6 each for the fat and lean lines) by sterile dissection following rapid decapitation, and then pooled together, respectively. Tissues were minced with scissors, then digested in 2 mg/ml collagenase type I (Invitrogen, Carlsbad, CA, USA) for 65 min at 37°C with shaking. Followed by filtration through a 20-μm screen and centrifugation at 300g for 10 min at room temperature, the pellets (preadipocytes) were suspended in the Trizol Reagent (Invitrogen, Carlsbad, CA, USA), and used directly for total RNAs extraction by the Trizol method following the manufacturer’s protocols (Invitrogen, Carlsbad, CA, USA).

### RNA sample preparation and sequencing

Subsequently, total RNAs were separated on a 15% polyacrylamide gel, and RNA molecules in the range of 18–30 nt were cut from the gel, extracted and ligated with appropriate adapters to the 5’ and 3’ termini. A reverse transcription reaction followed by PCR of low cycle number was performed to obtain sufficient products for Solexa sequencing (BGI, Shenzhen, China).

### Bioinformatics analyses

Raw short reads were passed through a quality control procedure, including trimming adaptors, discarding sequences shorter than 18 nt, eliminating low-quality sequences and adaptor-adaptor ligation, and removing all the repetitive and adaptor sequences. Data can be accessed at NCBI SRA archive (SRR1562981 and SRR1563007). Generated clean short reads were mapped onto the *G*. *gallus* genome (NCBI：GCA_000002315.2) using SOAP [99]. To identify already known miRNAs, perfectly matched short reads were then aligned to the *G*. *gallus* miRNA precursors deposited in the miRBase (release 20.0). The criteria used in the process were: 1) the unique sequence must be in perfect alignment with the precursor; 2) The start position of the alignment must be within +2 and-2 nt of the mature miRNA located in the precursor. To identify degenerated fragments of mRNA or other non-coding RNAs, such as rRNA, tRNA, and snoRNA, unique reads were screened against *G*. *gallus* non-coding RNA (excluding miRNA), and the annotated or predicted genes in the chicken reference genome (GeneBank and Rfam). Reads that matched perfectly with over 20 locations in the genome were removed.

The remaining short reads that could not be matched to known chicken miRNA precursors were searched against the metazoan mature miRNAs in the Sanger miRBase (20.0) [100], using the program Patscan to identify conserved miRNAs, with two mismatches allowed.

To find new miRNAs, we first removed those short reads sequenced less than twice in both libraries, and the remaining short reads were checked using the software Einverted of Emboss, to find step-loops or hairpin structures [101]. Secondly, an approach based on the characteristic hairpin structure of miRNA precursor was used to predict novel miRNAs. Mireap, developed by BGI of China (http://sourceforge.net/projects/mireap/), was used to perform the analysis, in which two constraints were used to detect novel miRNAs, the number of reads (>5), and the number of locations mapped onto these miRNAs in the chicken genome (<10) [91,102].

### Differentially expressed miRNA analysis

For each miRNA, numbers of reads were normalized to the total number of reads in each library, respectively. A web tool IDEG6 (http://telethon.bio.unipd.it/bioinfo/IDEG6/) was applied to detect differentially expressed miRNAs between the two chicken lines, and the Fisher’s exact test was used [103], which has been widely used in several other studies [104,105]. Circos was used to plot the differentially expressed miRNA, and read numbers of identified miRNAs in the two chicken lines as well [106].

### qRT-PCR verification of sequencing results

Primers for 15 known miRNAs were designed, according to a previously published method [107], to verify the sequencing results using quantitative RT-PCR method (S7 Table). From the fat and lean broiler lines at the 16^th^ generation, 5 chickens (14 days old) from each line were selected, respectively. For each animal, preadipocytes from the abdominal adipose tissue were collected, following the same procedure as described previously. Total RNA was extracted from these individual preadipocyte samples. A total of 1μg RNA for each sample was reverse-transcribed to cDNA using Reverse Transcription kits (TIANGEN Beijing, China). The real-time RT-PCR was performed on ABI 7500 system using SYBR Green PCR Kit from Takara (Dalian, China), with each miRNA checked in triplicate.

We selected 22 novel miRNAs for validation. According to their number of sequencing reads, these miRNAs were divided into three groups, high, medium and low. For each group, 4 miRNAs for each line were selected, respectively, which included 2 miRNAs common to both lines (S8 Table). Primers were designed for the regular stem-loop RT-PCR. The same RNA samples as used in miRNA sequencing were analyzed here again for novel miRNA validation. Kits for RNA reverse transcription and the real-time RT-PCR were from Promega (Madison, WI, USA) and Roche (Indianapolis, IN, USA), respectively.

The chicken small nuclear RNA U6 was used as the internal control, and the relative expression level of each miRNA was calculated using the 2^-ΔCt^ method, based on our experimental design. The t-test was used to determine the significance level of qRT-PCR expression level of 15 known miRNAs between the lean and fat chicken lines in the R statistical environment [108].

### Target gene prediction and gene ontology analysis

To predict genes targeted by miRNAs, TargetScan (http://www.targetscan.org/) was used [109], and the relationship between miRNA and mRNA interaction was analyzed and plotted by Cytoscape [110]. Gene ontology (GO) analyses of target genes were conducted by DAVID (http://david.abcc.ncifcrf.gov/) [111].

## Supporting Information

S1 Fig Linear regression analysis of gene expression from qRT-PCR and Solexa sequencing. (EPS)

S2 Fig Network relationship between miRNAs and their target genes significantly differentially expressed in chicken preadipocytes. miR-200b and miR-429 share the biggest number of common genes.
(EPS)

S1 Table Distribution of number of short reads for different types of RNAs (DOCX)

S2 Table Sequence similarity between miRNA precursors for the miRNA paralogs detected to be expressed in chicken preadipocytes. These miRNA paralogs have exactly the same mature miRNA sequences, and have already been detected and deposited in miRBase 20.0. Most of them are interchromosomal duplications, and can be divided into 3 groups according to their identity percentage between precusor sequences of miRNA paralogs. The first group are those perfectly matched to each other, the second group with >80% identity, and the third group with relatively low identity.
(DOC)

S3 Table Novel miRNAs discovered in the fat broiler line. Locations of miRNA in the chicken genome, their mature sequences, secondary structure, and counts of corresponding short reads were described.
(TXT)

S4 Table Novel miRNAs discovered in the lean broiler line. Locations of miRNA in the chicken genome, their mature sequences, secondary structure, and counts of corresponding short reads were described.
(TXT)

S5 Table MiRNAs reported previously to be potentially related to adipogenesis. (DOCX)

S6 Table Relationship between expression levels of miRNAs and their target genes. Up-regulated miRNAs target genes with lower expression levels, and *vice versa*. On average, 6% of all genes targeted by the 33 miRNAs between the two broiler lines follow this trend. These genes targeted by miRNAs were significantly differentially expressed in preadipocytes (unpublished data).
(DOCX)

S7 Table Information for primers used for 15 known miRNAs by qRT-PCR. (DOCX)

S8 Table Information for primers used for 22 novel miRNAs by qRT-PCR. (DOCX)
