# Supplementary material for: Expression Profiling of Preadipocyte MicroRNAs by Deep Sequencing on Chicken Lines Divergently Selected for Abdominal Fatness
Source: PLoS One. 2015 Feb 12;10(2):e0117843. doi: 10.1371/journal.pone.0117843 (PMC4326283; doi:10.1371/journal.pone.0117843)
Supplement: S1 Table — (DOCX) [file pone.0117843.s003.docx]

**Table S1**

| **Category** | **Lean line** | | **Fat line** | |
| --- | --- | --- | --- | --- |
|  | **Total sRNAs** | **Percentage** | **Total sRNAs** | **Percentage** |
| **exon_antisense** | 2695 | 0.02% | 2645 | 0.02% |
| **exon_sense** | 802750 | 5.96% | 369231 | 2.96% |
| **intron_antisense** | 18929 | 0.14% | 16039 | 0.13% |
| **intron_sense** | 58760 | 0.44% | 70713 | 0.57% |
| **miRNA** | 1155850 | 8.58% | 1055463 | 8.45% |
| **rRNA** | 4511236 | 33.51% | 5160927 | 41.32% |
| **repeat** | 28284 | 0.21% | 33877 | 0.27% |
| **scRNA** | 75488 | 0.56% | 41663 | 0.33% |
| **snRNA** | 166456 | 1.24% | 97321 | 0.78% |
| **snoRNA** | 56103 | 0.42% | 81239 | 0.65% |
| **tRNA** | 982047 | 7.29% | 1184824 | 9.49% |
| **unannotated** | 5605095 | 41.63% | 4376398 | 35.04% |
| **Total** | 13463693 | 100% | 12490340 | 100% |
