# Supplementary material for: Expression Profiling of Preadipocyte MicroRNAs by Deep Sequencing on Chicken Lines Divergently Selected for Abdominal Fatness
Source: PLoS One. 2015 Feb 12;10(2):e0117843. doi: 10.1371/journal.pone.0117843 (PMC4326283; doi:10.1371/journal.pone.0117843)
Supplement: S2 Table — These miRNA paralogs have exactly the same mature miRNA sequences, and have already been detected and deposited in miRBase 20.0. Most of them are interchromosomal duplications, and can be divided into 3 groups according to their identity percentage between precusor sequences of miRNA paralogs. The first group are those perfectly matched to each other, the second group with >80% identity, and the third group with relatively low identity. (DOC) [file pone.0117843.s004.doc]

| **Query id** | **Chromosome** | **Start** | **End** | **Subject id** | **Chromosome** | **Start** | **End** | **% identity** |
| --- | --- | --- | --- | --- | --- | --- | --- | --- |
| gga-mir-29b-1 | chr1 | 3328673 | 3328753 | gga-mir-29b-2 | chr26 | 2641616 | 2641695 | 83.95 |
| gga-let-7a-1 | chr1 | 71371153 | 71371174 | gga-let-7a-3 | chr24 | 3338940 | 3338961 | 89.87 |
| gga-let-7a-1 | chr1 | 71371153 | 71371174 | gga-let-7a-2 | chr12 | 6078445 | 6078466 | 82.89 |
| gga-miR-16-1 | chr1 | 168694548 | 168694631 | gga-miR-16-2 | chr9 | 21653410 | 21653503 | 60 |
| gga-mir-7-1 | chr10 | 12627325 | 12627346 | gga-mir-7b | chr28 | 4641066 | 4641087 | 95.19 |
| gga-mir-7-1 | chr10 | 12627325 | 12627346 | gga-mir-7-3 | chrZ | 39937052 | 39937073 | 84.44 |
| gga-miR-135a-1 | chr12 | 3003740 | 3003827 | gga-miR-135a-2 | chr1 | 46153508 | 46153607 | 61.39 |
| gga-miR-135a-1 | chr12 | 3003740 | 3003827 | gga-miR-135a-3 | chr26 | 2065494 | 2065589 | 58.33 |
| gga-mir-103-1 | chr13 | 4060404 | 4060481 | gga-mir-103-2 | chr4 | 88047783 | 88047865 | 77.11 |
| gga-mir-365-1 | chr14 | 766899 | 766983 | gga-mir-365-2 | chr18 | 6405040 | 6405135 | 73.96 |
| gga-mir-1698-1 | chr19 | 622426 | 622532 | gga-mir-1698-2 | chr19 | 687031 | 687137 | 100 |
| gga-mir-196-2 | chr2 | 32864721 | 32864741 | gga-mir-196-5 | chr27 | 3561349 | 3561369 | 100 |
| gga-mir-196-2 | chr2 | 32864721 | 32864741 | gga-mir-196-4 | chr2 | 33158255 | 33158275 | 100 |
| gga-mir-196-2 | chr2 | 32864721 | 32864741 | gga-mir-196-3 | chr2 | 32977484 | 32977504 | 83.13 |
| gga-miR-138-1 | chr2 | 41316138 | 41316233 | gga-miR-138-2 | chr11 | 2036243 | 2036325 | 55.34 |
| gga-mir-133a-1 | chr2 | 102176903 | 102176925 | gga-mir-133a-2 | chr20 | 8483509 | 8483531 | 96.51 |
| gga-mir-1813-1 | chr2 | 130687107 | 130687192 | gga-mir-1813-2 | chr10 | 16080572 | 16080645 | 30.23 |
| gga-mir-1a-1 | chr20 | 8472265 | 8472335 | gga-mir-1a-2 | chr2 | 102179979 | 102180063 | 70.59 |
| gga-miR-30c-1 | chr23 | 4884602 | 4884690 | gga-miR-30c-2 | chr3 | 81779099 | 81779170 | 58.89 |
| gga-miR-9-1 | chr28 | 3378847 | 3378934 | gga-miR-9-2 | chrZ | 60293370 | 60293456 | 75 |
| gga-miR-218-1 | chr4 | 74617807 | 74617915 | gga-miR-218-2 | chr13 | 3934522 | 3934616 | 65.14 |
| gga-mir-128-1 | chr7 | 30116396 | 30116477 | gga-mir-128-2 | chr2 | 46095826 | 46095909 | 71.43 |
| gga-mir-181a-1 | chr8 | 1986706 | 1986728 | gga-mir-181a-2 | chr17 | 9496926 | 9496948 | 91.67 |
| gga-mir-181b-1 | chr8 | 1986890 | 1986911 | gga-mir-181b-2 | chr17 | 9498558 | 9498579 | 86.11 |
| gga-mir-199-1 | chr8 | 4621404 | 4621426 | gga-mir-199-2 | chr17 | 5034565 | 5034587 | 89.33 |
| gga-mir-101-1 | chrZ | 27369524 | 27369602 | gga-mir-101-2 | chr8 | 27164028 | 27164103 | 70.89 |
