# Supplementary material for: Expression Profiling of Preadipocyte MicroRNAs by Deep Sequencing on Chicken Lines Divergently Selected for Abdominal Fatness
Source: PLoS One. 2015 Feb 12;10(2):e0117843. doi: 10.1371/journal.pone.0117843 (PMC4326283; doi:10.1371/journal.pone.0117843)
Supplement: S5 Table — (DOCX) [file pone.0117843.s007.docx]

**Table S5.**

| **Name** | **Function** | **Reference^※^** |
| --- | --- | --- |
| miR-33 | Very important in cholesterol homeostasis, regulating lipoprotein levels, and is used to develop molecular therapy | [16-24,112-133] |
| miR-206 | Suppression of LXR alpha-induced hepatic lipogenesis | [76] |
| miR-31 | Adipogenic differentiation in stem cells | [77] |
| miR-17-3p | Regulation of RB and fatty acyl desaturase expression | [9,66,67] |
| miR-429 | Upregulated in obese mice | [83,84] |
| miR-200b | Upregulated in obese mice | [83,84] |
| miR-1a | Suppression of LXR alpha-induced hepatic lipogenesis | [74] |
| miR-451 | Upregulated in coronary artery disease | [86] |
| miR-29b | Involvement in insulin resistance and T2D | [75,134-141] |
| miR-142-5p | Differentially expressed in adipose tissue when fed with high-fat diet | [79-81] |
| miR-200a | Upregulated in obese mice, impairing insulin and leptin signaling | [85] |
| miR-101 | Induction of 3T3-L1 differentiation, and up-regulated when fed with high-fat diet | [37,81] |
| miR-10a | Upregulated in hyperlipidemic sera | [82] |
| miR-142-3p | Increase in circulating miRNAs of T2D, and target the clock gene Bmal1 | [88,89] |
| miR-22* | Regulation of PTEN/AKT pathway and HDAC6 | [141-143] |
| miR-9 | Regulation of insulin secretion, and target acyl-coenzyme A:cholesterol acyltransferase-1 gene | [49, 144-150] |
| miR-15a | Target DLK1 and Foxo1, disrupt adipogenic differentiation and regulates insulin synthesis | [12,68-70] |
| miR-32 | Lipid metabolism in oligodendrocytes and myelin | [78] |
| miR-218 | Perturbed expression in the hypothalamus after metabolic stress | [87] |
| miR-222 | Target kit ligand, affect adipose tissue development and involved in diabetes | [34,151] |
| miR-454 | Decrease hepatic cytochrome P450 3A activity in cirrhosis | [90] |

Note: **^※^** indicates additional references are listed as follows.

**References:**

1. Allen RM, Marquart TJ, Albert CJ, Suchy FJ, Wang DQ, et al.(2012) miR-33 controls the expression of biliary transporters, and mediates statin- and diet-induced hepatotoxicity. EMBO Mol Med. 4:882-895.
2. Allen RM, Marquart TJ, Jesse JJ, Baldán A (2014) Control of Very Low-Density Lipoprotein Secretion by N-Ethylmaleimide-Sensitive Factor and miR-33. Circ Res. 115:10-22.
3. Canfrán-Duque A, Ramírez CM, Goedeke L, Lin CS, Fernández-Hernando C (2014) microRNAs and HDL life cycle. Cardiovasc Res. pii: cvu140.
4. Chen J, Smith LE (2013) Altered cholesterol homeostasis in aged macrophages linked to neovascular macular degeneration. Cell Metab. 17:471-472.
5. Cirera-Salinas D, Pauta M, Allen RM, Salerno AG, Ramírez CM, et al. (2012) Mir-33 regulates cell proliferation and cell cycle progression. Cell Cycle 11:922-933.
6. Dávalos A, Fernández-Hernando C (2013) From evolution to revolution: miRNAs as pharmacological targets for modulating cholesterol efflux and reverse cholesterol transport. Pharmacol Res. 75:60-72.
7. Fernández-Hernando C, Moore KJ (2011) MicroRNA modulation of cholesterol homeostasis. Arterioscler Thromb Vasc Biol. 31:2378-2382.
8. Fernández-Hernando C, Suárez Y, Rayner KJ, Moore KJ (2011) MicroRNAs in lipid metabolism. Curr Opin Lipidol. 22:86-92.
9. Goedeke L, Vales-Lara FM, Fenstermaker M, Cirera-Salinas D, Chamorro-Jorganes A, et al. (2013) A regulatory role for microRNA 33* in controlling lipid metabolism gene expression. Mol Cell Biol. 33:2339-2352.
10. Hicks JA, Trakooljul N, Liu HC (2010) Discovery of chicken microRNAs associated with lipogenesis and cell proliferation. Physiol Genomics. 41:185-193.
11. Ho PC, Chang KC, Chuang YS, Wei LN (2011) Cholesterol regulation of receptor-interacting protein 140 via microRNA-33 in inflammatory cytokine production. FASEB J. 25:1758-1766.
12. Horie T, Baba O, Kuwabara Y, Chujo Y, Watanabe S, et al. (2012) MicroRNA-33 deficiency reduces the progression of atherosclerotic plaque in ApoE-/- mice. J Am Heart Assoc. 1:e003376.
13. Horie T, Nishino T, Baba O, Kuwabara Y, Nakao T, et al. (2013) MicroRNA-33 regulates sterol regulatory element-binding protein 1 expression in mice. Nat Commun. 4:2883.
14. Horie T, Ono K, Horiguchi M, Nishi H, Nakamura T, et al. (2010) MicroRNA-33 encoded by an intron of sterol regulatory element-binding protein 2 (Srebp2) regulates HDL in vivo. Proc Natl Acad Sci U S A. 107:17321-17326.
15. Marquart TJ, Allen RM, Ory DS, Baldán A (2010) miR-33 links SREBP-2 induction to repression of sterol transporters. Proc Natl Acad Sci U S A. 107:12228-12232.
16. Marquart TJ, Wu J, Lusis AJ, Baldán Á (2013) Anti-miR-33 therapy does not alter the progression of atherosclerosis in low-density lipoprotein receptor-deficient mice. Arterioscler Thromb Vasc Biol. 33:455-458.
17. Moore KJ, Rayner KJ, Suárez Y, Fernández-Hernando C (2011) The role of microRNAs in cholesterol efflux and hepatic lipid metabolism. Annu Rev Nutr. 31:49-63.
18. Moore KJ, Rayner KJ, Suárez Y, Fernández-Hernando C (2010). microRNAs and cholesterol metabolism. Trends Endocrinol Metab. 21:699-706.
19. Norata GD, Sala F, Catapano AL, Fernández-Hernando C (2013) MicroRNAs and lipoproteins: a connection beyond atherosclerosis? Atherosclerosis 227:209-215.
20. Rottiers V, Obad S, Petri A, McGarrah R, Lindholm MW, et al. (2013) Pharmacological inhibition of a microRNA family in nonhuman primates by a seed-targeting 8-mer antimiR. Sci Transl Med. 5:212ra162.
21. Sene A, Khan AA, Cox D, Nakamura RE, Santeford A, et al. (2013) Impaired cholesterol efflux in senescent macrophages promotes age-related macular degeneration. Cell Metab. 17:549-561.
22. Hu Y, Zhang R, Zhang Y, Li J, Grossmann R, et al. (2012) In ovo leptin administration affects hepatic lipid metabolism and microRNA expression in newly hatched broiler chickens. J Anim Sci Biotechnol. 3:16.
23. Peng H, Zhong M, Zhao W, Wang C, Zhang J, et al. (2013) Urinary miR-29 Correlates with Albuminuria and Carotid Intima-Media Thickness in Type 2 Diabetes Patients. PLoS One 8: e82607.
24. Liang J, Liu C, Qiao A, Cui Y, Zhang H, et al. (2013) MicroRNA-29a-c decrease fasting blood glucose levels by negatively regulating hepatic gluconeogenesis. J Hepatol. 58:535-542.
25. Maegdefessel L, Azuma J, Toh R, Merk DR, Deng A, et al. (2012) Inhibition of microRNA-29b reduces murine abdominal aortic aneurysm development. J Clin Invest. 122:497-506.
26. Merk DR, Chin JT, Dake BA, Maegdefessel L, Miller MO, et al. (2012) miR-29b participates in early aneurysm development in Marfan syndrome. Circ Res. 110:312-324.
27. Boon RA, Seeger T, Heydt S, Fischer A, Hergenreider E, et al. (2011) MicroRNA-29 in aortic dilation: implications for aneurysm formation. Circ Res. 109:1115-1119.
28. Pullen TJ, da Silva Xavier G, Kelsey G, Rutter GA (2011) miR-29a and miR-29b contribute to pancreatic beta-cell-specific silencing of monocarboxylate transporter 1 (Mct1). Mol Cell Biol. 31:3182-3194.
29. Chen HY, Zhong X, Huang XR, Meng XM, You Y, et al. (2014) MicroRNA-29b inhibits diabetic nephropathy in db/db mice. Mol Ther. 22:842-853.
30. Xu J, Hu G, Lu M, Xiong Y, Li Q, et al. (2013) MiR-9 reduces human acyl-coenzyme A:cholesterol acyltransferase-1 to decrease THP-1 macrophage-derived foam cell formation. Acta Biochim Biophy Sin 45: 953-962.
31. Plaisance V, Abderrahmani A, Perret-Menoud V, Jacquemin P, Lemaigre F, et al. (2006) MicroRNA-9 controls the expression of Granuphilin/Slp4 and the secretory response of insulin-producing cells. J Biol Chem. 281:26932-26942.
32. Setyowati Karolina D, Sepramaniam S, Tan HZ, Armugam A, Jeyaseelan K (2013) miR-25 and miR-92a regulate insulin I biosynthesis in rats. RNA Biol. 10:1365-1378.
33. Chakraborty C, George Priya Doss C, Bandyopadhyay S (2013) miRNAs in insulin resistance and diabetes-associated pancreatic cancer: the 'minute and miracle' molecule moving as a monitor in the 'genomic galaxy'. Curr Drug Targets. 14:1110-1117.
34. Ramachandran D, Roy U, Garg S, Ghosh S, Pathak S, et al. (2011) Sirt1 and mir-9 expression is regulated during glucose-stimulated insulin secretion in pancreatic β-islets. FEBS J. 278:1167-1174.
35. Joglekar MV, Joglekar VM, Hardikar AA (2009) Expression of islet-specific microRNAs during human pancreatic development. Gene Expr Patterns. 9:109-113.
36. Bye A, Rosjo H, Aspenes ST, Condorelli G, Omland T, et al. (2013) Circulating microRNAs and aerobic fitness--the HUNT-Study. PLoS One. 8:e57496.
37. Parra P, Serra F, Palou A (2010) Expression of adipose microRNAs is sensitive to dietary conjugated linoleic acid treatment in mice. PLoS One. 5:e13005.
38. Shi Z, Zhao C, Guo X, Ding H, Cui Y, et al. (2014) Differential expression of microRNAs in omental adipose tissue from gestational diabetes mellitus subjects reveals miR-222 as a regulator of ERα expression in estrogen-induced insulin resistance. Endocrinology. 155:1982-1990.
39. Zhao C, Dong J, Jiang T, Shi Z, Yu B, et al. (2011) Early second-trimester serum miRNA profiling predicts gestational diabetes mellitus. PLoS One. 6:e23925.
40. Herrera BM, Lockstone HE, Taylor JM, Ria M, Barrett A, et al. (2010) Global microRNA expression profiles in insulin target tissues in a spontaneous rat model of type 2 diabetes. Diabetologia. 53:1099-1109.
