# Supplementary material for: Expression Profiling of Preadipocyte MicroRNAs by Deep Sequencing on Chicken Lines Divergently Selected for Abdominal Fatness
Source: PLoS One. 2015 Feb 12;10(2):e0117843. doi: 10.1371/journal.pone.0117843 (PMC4326283; doi:10.1371/journal.pone.0117843)
Supplement: S6 Table — Up-regulated miRNAs target genes with lower expression levels, and vice versa. On average, 6% of all genes targeted by the 33 miRNAs between the two broiler lines follow this trend. These genes targeted by miRNAs were significantly differentially expressed in preadipocytes (unpublished data). (DOCX) [file pone.0117843.s008.docx]

**Table S6.**

| **microRNA** | **Expression level in the fat broiler line** | **No. of target genes predicted** | **No. of target genes with the same trend^#^** | **Percentage** |
| --- | --- | --- | --- | --- |
| **miR-1b** | down-regulated | 285 | 15 | 0.05 |
| **miR-32** | down-regulated | 504 | 34 | 0.06 |
| **miR-218** | down-regulated | 332 | 27 | 0.08 |
| **miR-222** | down-regulated | 603 | 19 | 0.03 |
| **miR-454** | down-regulated | 462 | 40 | 0.08 |
| **miR-460a** | down-regulated | 142 | 6 | 0.04 |
| **miR-1416** | down-regulated | 48 | 0 | 0 |
| **miR-1563** | up-regulated | 105 | 8 | 0.07 |
| **miR-1a** | up-regulated | 285 | 30 | 0.11 |
| **miR-9** | up-regulated | 385 | 30 | 0.08 |
| **miR-15a** | up-regulated | 460 | 28 | 0.06 |
| **miR-17-3p** | up-regulated | 114 | 6 | 0.05 |
| **miR-22** | up-regulated | 112 | 9 | 0.08 |
| **miR-29b** | up-regulated | 260 | 22 | 0.08 |
| **miR-31** | up-regulated | 79 | 4 | 0.05 |
| **miR-33** | up-regulated | 188 | 7 | 0.04 |
| **miR-101** | up-regulated | 478 | 35 | 0.07 |
| **miR-142-3p** | up-regulated | 118 | 8 | 0.07 |
| **miR-142-5p** | up-regulated | 159 | 7 | 0.04 |
| **miR-200a** | up-regulated | 273 | 21 | 0.08 |
| **miR-200b** | up-regulated | 458 | 37 | 0.08 |
| **miR-206** | up-regulated | 285 | 0 | 0 |
| **miR-429** | up-regulated | 458 | 37 | 0.08 |
| **miR-451** | up-regulated | 6 | 0 | 0 |
| **miR-1306** | up-regulated | 2 | 1 | 0.5 |
| **miR-1434** | up-regulated | 24 | 1 | 0.04 |
| **miR-1551** | up-regulated | 215 | 11 | 0.05 |
| **miR-1653** | up-regulated | 24 | 1 | 0.04 |
| **miR-1684** | up-regulated | 43 | 0 | 0 |
| **miR-1805-3p** | up-regulated | 46 | 0 | 0 |
| **miR-2188** | up-regulated | 33 | 3 | 0.09 |
| **miR-3535** | up-regulated | 85 | 8 | 0.09 |
| **miR-10a*** | up-regulated | 0 | 0 | - |

Note: ^#^ indicates genes predicted to be targeted by miRNAs down-regulated in the fat chicken line have higher expression levels, and *vice versa*.
