# Supplementary material for: Expression Profiling of Preadipocyte MicroRNAs by Deep Sequencing on Chicken Lines Divergently Selected for Abdominal Fatness
Source: PLoS One. 2015 Feb 12;10(2):e0117843. doi: 10.1371/journal.pone.0117843 (PMC4326283; doi:10.1371/journal.pone.0117843)
Supplement: S7 Table — (DOCX) [file pone.0117843.s009.docx]

**Table S7.**

|  | **Primer 5'to 3'** |
| --- | --- |
| **RT** | GTGTCGTGGAGTCGGCAACAGTTGAGTTTTTTTTTTTTTTTTTTVN |
| **URP** | GTCGTGGAGTCGGCAACAGTTG |
| **U6-F** | CACGCAAATTCGTGAAGCGTTCCA |
| **miR-92-F** | TATTGCACTTGTCCCGGCCTG |
| **miR-458-F** | GCGCATAGCTCTTTGAATGGTACTGC |
| **miR-221-F** | GGAGCTACATTGTCTGCTGGGTTTC |
| **miR-2188-F** | GAAGGTCCAACCTCACATGTCCT |
| **miR-21-F** | GCCGGTAGCTTATCAGACTGATGTTG |
| **miR-1A-F** | CCCGTGGAATGTAAAGAAGTATGTA |
| **miR-19A-F** | GGGTGTGCAAATCTATGCAAAACTGA |
| **miR-181b-F** | GGAACATTCATTGCTGTCGGTGGG |
| **miR-17-3p-F** | ACTGCAGTGAAGGCACTTGT |
| **miR-148A-F** | CCCTCAGTGCACTACAGAACTTTGT |
| **miR-146A-F** | GCTGAGAACTGAATTCCATGGGTT |
| **miR-130a-F** | CGGCAGTGCAATATTAAAAGGGCAT |
| **miR-103-F** | CGAGCAGCATTGTACAGGGCTATG |
| **miR-101-F** | CGTACAGTACTGTGATAACTGAA |
| **miR-100-F** | CCGAACCCGTAGATCCGAACTTGTG |
