# Supplementary material for: Expression Profiling of Preadipocyte MicroRNAs by Deep Sequencing on Chicken Lines Divergently Selected for Abdominal Fatness
Source: PLoS One. 2015 Feb 12;10(2):e0117843. doi: 10.1371/journal.pone.0117843 (PMC4326283; doi:10.1371/journal.pone.0117843)
Supplement: S8 Table — (DOCX) [file pone.0117843.s010.docx]

**Table S8**

| **Name** | **Read number (level)** | **Primer 5'to 3'** | **True** |
| --- | --- | --- | --- |
| I-0252 | 220 (high) | RT: CTCAACTGGTGTCGTGGAGTCGGCAATTCAGTTGAGAAGTGCAT | yes |
|  |  | F: ACACTCCAGCTGGGACTGAACCTGG |  |
| I-0199 | 155 (high) | RT: CTCAACTGGTGTCGTGGAGTCGGCAATTCAGTTGAGAGGTCTCA | yes |
|  |  | F: ACACTCCAGCTGGGTGACTGATAGAAGC |  |
| I-0194 | 68 (high) | RT: CTCAACTGGTGTCGTGGAGTCGGCAATTCAGTTGAGCGGCTTCT | yes |
|  |  | F: ACACTCCAGCTGGGCATGGAGAGCACTGA |  |
| I-0276 | 66 (high) | RT: CTCAACTGGTGTCGTGGAGTCGGCAATTCAGTTGAGTCCCCTCC | yes |
|  |  | F: ACACTCCAGCTGGGTTGTGAAACTGG |  |
| I-0056 | 52 (medium) | RT: CTCAACTGGTGTCGTGGAGTCGGCAATTCAGTTGAGCCCGTCAT | yes |
|  |  | F: ACACTCCAGCTGGGAAGGAGCTGGAG |  |
| I-0182 | 47 (medium) | F: CTCAACTGGTGTCGTGGAGTCGGCAATTCAGTTGAGCCTTCCTC | yes |
|  |  | R: ACACTCCAGCTGGGATAGGGAGGTAG |  |
| I-0426 | 37 (medium) | RT: CTCAACTGGTGTCGTGGAGTCGGCAATTCAGTTGAGGACACCAC | yes |
|  |  | F: ACACTCCAGCTGGGATGCTGTGGAGC |  |
| I-0094 | 23 (low) | RT: CTCAACTGGTGTCGTGGAGTCGGCAATTCAGTTGAGCGGTCCAC | yes |
|  |  | F: ACACTCCAGCTGGGAGCGGAGCTGTG |  |
| I-0254 | 20 (low) | RT: CTCAACTGGTGTCGTGGAGTCGGCAATTCAGTTGAGGGTACCAG | yes |
|  |  | F: ACACTCCAGCTGGGAAGACGTGATCG |  |
| I-0038 | 10 (low) | RT: CTCAACTGGTGTCGTGGAGTCGGCAATTCAGTTGAGGGCTCCCA | yes |
|  |  | F: ACACTCCAGCTGGGAGGATGCTGAGG |  |
| I-0188 & II-0689 | 57 (medium) & 12 (low) | RT: CTCAACTGGTGTCGTGGAGTCGGCAATTCAGTTGAGGCCTTCTC | yes |
|  |  | F: ACACTCCAGCTGGGGACGGACCGGGAGAA |  |
| I-0016 & II-0059 | 16 (low) & 11 (low) | RT: CTCAACTGGTGTCGTGGAGTCGGCAATTCAGTTGAGATCACTCC | no |
|  |  | F: ACACTCCAGCTGGGGGAGATTGTCC |  |
| II-0414 | 86 (high) | RT: CTCAACTGGTGTCGTGGAGTCGGCAATTCAGTTGAGCAAAATAA | yes |
|  |  | F: ACACTCCAGCTGGGTTCTGGAGGAGG |  |
| II-0602 | 76 (high) | RT: CTCAACTGGTGTCGTGGAGTCGGCAATTCAGTTGAGTACCTTCC | no |
|  |  | F: ACACTCCAGCTGGGTCTGGGATGCAG |  |
| II-1005 | 68 (high) | RT: CTCAACTGGTGTCGTGGAGTCGGCAATTCAGTTGAGGCCTCCAA | yes |
|  |  | F: ACACTCCAGCTGGGTGAGATGAGAGCTGC |  |
| II-0634 | 61 (high) | RT: CTCAACTGGTGTCGTGGAGTCGGCAATTCAGTTGAGTCATTCTT | no |
|  |  | F: ACACTCCAGCTGGGCACAAGGACGAT |  |
| II-0674 | 46 (medium) | RT: CTCAACTGGTGTCGTGGAGTCGGCAATTCAGTTGAGACCGCTTC | no |
|  |  | F: ACACTCCAGCTGGGTTTCTGTCCCAGCTG |  |
| II-0105 | 41 (medium) | RT: CTCAACTGGTGTCGTGGAGTCGGCAATTCAGTTGAGCTAACAGT | no |
|  |  | F: ACACTCCAGCTGGGAGAGAAGAGAG |  |
| II-1249 | 35 (medium) | RT: CTCAACTGGTGTCGTGGAGTCGGCAATTCAGTTGAGGCTCCCTT | yes |
|  |  | F: ACACTCCAGCTGGGTGACCGGAGCC |  |
| II-0766 | 30 (medium) | RT: CTCAACTGGTGTCGTGGAGTCGGCAATTCAGTTGAGGCCCCGCG | yes |
|  |  | F: ACACTCCAGCTGGGCGGACAGGTGGC |  |
| II-0166 | 15 (low) | RT: CTCAACTGGTGTCGTGGAGTCGGCAATTCAGTTGAGGCTCCTGC | yes |
|  |  | F: ACACTCCAGCTGGGGGTGGTCGTCAAC |  |
| II-0731 | 10 (low) | RT: CTCAACTGGTGTCGTGGAGTCGGCAATTCAGTTGAGAGTTCCTG | yes |
|  |  | F: ACACTCCAGCTGGGAAGCGGGCTTTG |  |
| U6 |  | F: CTCGCTTCGGCAGCACA |  |
|  |  | R: AACGCTTCACGAATTTGCGT |  |
| loop-URP |  | TGGTGTCGTGGAGTCG |  |

Note: RT indicates primers used for reverse transcription, and U6R as well. Primers indicated with F, and universal reverse primer (loop-URP) were used for QRT-PCR. Primers U6F and U6R were used to amplify the U6 RNA.
